# *Helicobacter pylori* Outer Membrane Vesicle Size Determines Their Mechanisms of Host Cell Entry and Protein Content

**DOI:** 10.3389/fimmu.2018.01466

**Published:** 2018-07-02

**Authors:** Lorinda Turner, Natalie J. Bitto, David L. Steer, Camden Lo, Kimberley D’Costa, Georg Ramm, Mitch Shambrook, Andrew F. Hill, Richard L. Ferrero, Maria Kaparakis-Liaskos

**Affiliations:** ^1^Centre for Innate Immunity and Infectious Diseases, Hudson Institute of Medical Research, Clayton, Melbourne, VIC, Australia; ^2^Department of Physiology, Anatomy and Microbiology, School of Life Sciences, La Trobe University, Melbourne, VIC, Australia; ^3^Research Centre for Extracellular Vesicles, School of Molecular Sciences, La Trobe University, Melbourne, VIC, Australia; ^4^Monash University, Clayton, VIC, Australia; ^5^Monash Micro Imaging, Monash University, Clayton, VIC, Australia; ^6^Monash Biomedical Proteomics Facility, Monash University, Clayton, VIC, Australia; ^7^Department of Biochemistry and Molecular Biology, Monash University, Melbourne, VIC, Australia; ^8^La Trobe Institute for Molecular Sciences, La Trobe University, Melbourne, VIC, Australia; ^9^Department of Microbiology, Biomedicine Discovery Institute, Monash University, Melbourne, VIC, Australia

**Keywords:** bacterial membrane vesicles, endocytosis, macropinocytosis, pathogenesis, proteomics, outer membrane vesicles, size

## Abstract

Gram-negative pathogens ubiquitously shed outer membrane vesicles (OMVs) that play a central role in initiating and regulating pathogenesis in the host. Due to their highly inflammatory nature, OMVs are extensively being examined for their role in mediating disease in addition to their applications in innovative vaccines. A key mechanism whereby OMVs mediate inflammation and disease progression is dependent on their ability to enter host cells. Currently, the role of OMV size on determining their mechanism of cellular entry and their protein composition remains unknown. In this study, we examined the mechanisms whereby OMV size regulates their mode of entry into epithelial cells, in addition to their protein cargo and composition. We identified that a heterogeneous sized population of *Helicobacter pylori* OMVs entered epithelial cells *via* macropinocytosis, clathrin, and caveolin-dependent endocytosis. However, smaller OMVs ranging from 20 to 100 nm in size preferentially entered host cells *via* caveolin-mediated endocytosis. Whereas larger OMVs ranging between 90 and 450 nm in size entered host epithelial cells *via* macropinocytosis and endocytosis. Most importantly, we identified the previously unknown contribution that OMV size has on determining their protein content, as fewer and less diverse bacterial proteins were contained within small OMVs compared to larger OMVs. Collectively, these findings identify the importance of OMV size in determining the mechanisms of OMV entry into host cells, in addition to regulating their protein cargo, composition, and subsequent immunogenicity. These findings have significant implications in broadening our understanding of the bacterial regulation of virulence determinants and immunogenic proteins associated with OMVs, their role in mediating pathogenesis and in refining the design and development of OMV-based vaccines.

## Introduction

Gram-negative bacteria ubiquitously shed vesicles known as outer membrane vesicles (OMVs) during their normal growth [reviewed in Ref. (1, 2)]. OMVs are spherical, bi-layered membrane vesicles ranging from approximately 20 to 350 nm in size, and their release occurs naturally both *in vitro* and *in vivo*. The importance of OMV production during the natural course of infection and in pathogenesis has been highlighted by the identification of OMVs within infected host tissues, including the gastric mucosa of *Helicobacter pylori* infected individuals (3), as well as in the cerebrospinal fluid and sera of patients with meningococcal infection (4). In addition, the ability of OMVs produced by commensal bacteria to prevent diseases such as experimental colitis has been reported (5), further broadening the role of OMVs in disease and gut homeostasis.

Outer membrane vesicles from a range of bacteria have been identified to have a similar protein (6–8) and lipid (9) composition to the outer membranes of their parent bacterium. Specifically, OMVs may contain inner and outer membrane proteins, periplasmic proteins (10), lipopolysaccharide (LPS) (10), peptidoglycan (PG) (11), DNA (12–14), and toxins (3, 15–18). As the protein composition of OMVs is highly similar to that of their parent bacterium, their use and development as innovative vaccines is being extensively examined (19–27). Therefore, due to the similarity of OMVs to their parent bacterium and their highly immunogenic nature, OMV-based vaccines are currently being developed and licensed for human use [reviewed in Ref. (27, 28)].

As OMVs contain many pathogenic proteins originating from their parent bacterium, they are extremely effective at initiating and regulating pro-inflammatory responses in the host. For example, OMVs from the Gram-negative pathogens *H. pylori, Neisseria, Pseudomonas, Campylobacter*, and *Vibrio* induce the secretion of interleukin-8 (IL-8) by non-phagocytic epithelial cells (11, 29–31). The ability of OMVs to initiate and mediate a pro-inflammatory response in host epithelial cells is largely dependent upon their uptake and entry into host cells. There are numerous reported mechanisms, whereby OMVs enter non-phagocytic epithelial cells to mediate inflammation in the host. These include lipid-raft-dependent (11, 32–35), or lipid-raft-independent mechanisms (29), in addition to the requirement for endocytosis (32, 34, 36–38) or macropinocytosis (33). However, to date, the contribution of OMV size on determining the mechanism of OMV entry into non-phagocytic epithelial cells, in addition to determining their protein composition has not been examined and is the focus of this work.

In this study, we characterized the mechanisms whereby *H. pylori* OMV size regulates their route of endocytic entry into non-phagocytic epithelial cells, in addition to regulating their protein content. Our findings revealed that a heterogeneous sized population of OMVs entered human epithelial cells *via* macropinocytosis, caveolin, and clathrin-dependent endocytosis. We identified the previously unknown contribution of OMV size on determining the mechanism of entry into host cells. Specifically, we found that smaller *H. pylori* OMVs ranging from 20 to 100 nm in size entered epithelial cells *via* macropinocytosis, clathrin, and caveolin-dependent endocytosis, and that inhibition of caveolin had the greatest reduction in small OMV entry into host cells. However, the entry of larger OMVs into epithelial cells was inhibited by all mechanisms of endocytosis and did not appear to display a bias for entry *via* any particular mechanism. Most importantly, we determined that OMV size predetermines the protein composition of OMVs, as larger OMVs contain a greater number and wider range of proteins when compared to smaller OMVs. Collectively these findings are the first to report that OMV size plays a role in the mechanisms of host cell entry and their protein content and composition. These findings have major implications for understanding the role of OMVs in mediating bacterial pathogenesis and facilitating their design and development as innovative vaccines.

## Materials and Methods

### Bacterial Strains and OMV Purification

*Helicobacter pylori* 251 *cag*PAI (11) was cultured using Horse Blood Agar medium (Blood Agar Base No2, Oxoid) or in Brain Heart Infusion broth (Becton Dickinson, USA), supplemented with 0.6% (w/v) β-cyclodextrin (Sigma-Aldrich, USA) by shaking at 120 rpm. Cultures were grown at 37°C under microaerobic conditions. *H. pylori* OMVs were purified from log phase cultures as described previously (11). In brief, bacteria were pelleted from overnight cultures by centrifugation at 2,500 × *g* for 20 min. Supernatants were subsequently filtered using a 0.22 µm PES filter and OMVs were pelleted from these supernatants by ultracentrifugation (100,000 × *g*, 2 h, 4°C). The resulting OMV pellets were resuspended in PBS and protein concentrations determined by the Bradford Protein Assay (Bio-Rad, USA).

### Separation of OMVs by Size Using Sucrose Gradient Purification

Outer membrane vesicle preparations in 6 ml were layered onto discontinuous sucrose gradients, consisting of 12.5 ml 25% (w/v) sucrose, 15.5 ml 42% (w/v) sucrose, and 5 ml 56% (w/v) sucrose and subjected to ultracentrifugation (100,000 × *g*, 16 h, 4°C) (11). Thirteen fractions (3 ml each) were collected, washed with PBS to remove any remaining sucrose, and concentrated to a final volume of 500 µl using Amicon YM-10 columns (Millipore, Ireland).

### Fluorescent Labeling of OMVs

Outer membrane vesicles (2 mg/ml) were labeled with 1% (v/v) 3,3′-dioctadecyloxacarbocyanine perchlorate (DiO; Molecular Probes, USA) for 20 min at 37°C (39). Excess dye was removed by washing OMVs three times with PBS using a 10 kDa MWCO filtration column (Amicon).

### Cell Culture

Human gastric adenocarcinoma (AGS) and human embryonic kidney (HEK293) cells were routinely cultured using RPMI or DMEM respectively, supplemented with 10% (v/v) fetal calf serum (FCS). Cells were seeded at a density of 1 × 10^5^ cells per ml in 12- or 24-well plate for 24 h. For IL-8 secretion studies, cells were co-cultured with heterogeneous, sucrose purified small or large OMVs (50 µg/ml) for 24 h. IL-8 in cultured supernatants was quantified using the BD OptEIA human IL-8 ELISA kit as per the manufacturer’s instructions (BD Biosciences, USA).

### Chemical Inhibition of OMV Entry into Epithelial Cells

Inhibition of OMV entry was performed using chemical inhibitors of endocytosis (all from Sigma-Aldrich, USA) at the following concentrations, as described previously (40): cytochalasin D (2 µM), dynasore monohydrate (10 µM), nocodazole (3.3 µM), valinomycin (10 µM), or chlorpromazine (15 µg/ml) (29). AGS or HEK293 cells were pre-treated with inhibitors for 30 min. The cells were subsequently washed twice and the media replaced prior to incubation with 50 µg/ml of OMVs for 4 or 16 h for fluorescence analysis and 24 h to quantify IL-8 production by ELISA.

### Cytotoxicity Assay

AGS or HEK293 cells were treated with endocytosis inhibitors or 0.5% (w/v) sodium azide (41) for 30 min. Cells were then washed and media replaced for 4 h. Cellular cytotoxicity was measured using the CellTiter-Glo^®^ assay (Promega, USA), according to the manufacturer’s instructions. Luminescence was measured using a FLUOstar OPTIMA (BMG Labtech, Australia).

### siRNA Knockdown and qRT-PCR

siRNA knockdown and validation of knockdown was performed as previously described (39). In brief, AGS cells were transfected with two pre-designed and inventoried siRNAs at a final concentration of 10 nM, using Lipofectamine 2000 (Invitrogen). The siRNA sequences used were: *PAK1* (s10019, s10021), *DNM2* (s4212, s4213), *CAV1* (s2447, s2448), and *CLTC* (s477, s475) (Ambion, Applied Biosystems). As a control, cells were transfected with control siRNA (Qiagen, VIC, Australia). To determine the effectiveness of siRNA knockdown, RNA was isolated from siRNA transfected AGS cells using the Purelink RNA mini kit (Life Technologies) and reverse transcribed into cDNA using Superscript III and oligo (dT) primers (Life Technologies). Gene silencing was assessed by TaqMan qRT-PCR using validated FAM labeled *PAK1* (Hs00945621_m1), *DNM2* (Hs00974698_m1), *CAV1* (Hs00971716_m1), and *CLTC* (Hs00964504_m1) primers (all from Ambion, Applied Biosystems), and 18S rRNA FAM labeled primer sets (assay, ID 4319413E, Applied Biosystems). Assays were performed in triplicate using MicroAmp Optical 384-well reaction plate (Applied Biosystems). Target gene cDNA concentrations for each sample were determined using the standard curve and normalized to 18S rRNA expression.

### Flow Cytometry

The effectiveness of trypan blue quenching of OMV-associated DiO fluorescence was examined by flow cytometry. AGS cells were incubated with DiO labeled OMVs for 4 h prior to permeabilization with 0.01% (v/v) Triton-X for 10 min, or not permeabilized. Fluorescence was quenched with trypan blue (0.025% final concentration). Cells were washed once and resuspended in DPBS (Gibco, Invitrogen, NY, USA) containing 2% (v/v) FCS and analyzed by flow cytometry using BD FACS CANTO II and BD FACS Diva software v6.0. A total of 6 × 10^4^ cells were counted for each condition. Data were analyzed using FlowJo version 7.6.

### Fluorescence Microscopy

AGS cells were seeded onto glass coverslips in 12-well plate (Becton Dickinson Labware, NJ, USA) and cultured overnight. Cells were pre-treated with inhibitors for 30 min, washed twice and media replaced, prior to co-culture with 50 µg/ml of DiO labeled OMVs for 4 or 16 h as indicated. Effectiveness of trypan blue quenching of DiO fluorescence was examined by permeabilization of cells with 0.01% (v/v) Triton-X for 10 min, prior to addition of trypan blue. For all other experiments, extracellular fluorescence was quenched using 0.025% (v/v) trypan blue (Sigma Chemical Co., MO, USA) (29), prior to washing three times with PBS and fixing with 4% (v/v) formaldehyde (Merck, Darmstadt, Germany) for 20 min. Nuclei were stained with 4′,6-diamidino-2-phenylindole, dilactate (DAPI; Molecular Probes, OR, USA), and mounted in Dako Fluorescent mounting medium (Dako North America Inc., CA, USA). To confirm the effectiveness of chemical inhibition, cells were pre-treated with inhibitors followed by the addition of either pHRODO red conjugated human transferrin (hTf; 16.7 µg/ml) (42) or FITC conjugated Dextran, 70 kDa (Dex70; 2.5 mg/ml) (43), for 4 h. Extracellular fluorescence was quenched with trypan blue and cells were fixed and mounted as described previously. Images were acquired on an Applied Precision Instruments DeltaVision deconvolution microscope using a 40 × 1.35NA oil objective at 512 × 512 × 14 bit per channel. Z-stacks (10–15 µm) at 0.2 µm per slice were acquired and deconvolved based on the point spread function of the system. Images were analyzed by Imaris (v7.1.0 Bitplane AG), where the average intensity density of OMV fluorescence was derived by measuring the sum intensity of OMV fluorescence multiplied by the OMV volume, then averaged across cells in the field of view. These arbitrary intensity density units were then normalized to OMV alone groups and expressed as average signal density. For inhibition analysis, the means of each condition were determined for three-independent experiments and plotted as average signal density.

### Examination of OMVs by Transmission Electron Microscopy and NanoSight

Outer membrane vesicle samples were prepared for electron microscopy as described previously (44). Grids were viewed using a Hitachi H-7500 transmission electron microscope at 70 K × view and images captured using Digital micrograph™ 1.71.38 (Gatan Inc.). Image analysis was performed using ImageJ v1.47n. OMV size was determined using NanoSight NTA 3.2 (Malvern Instruments, UK). NanoSight particle tracking analysis was performed using heterogeneous OMVs in addition to sucrose gradient purified OMVs obtained from fractions 6 and 12. Fractions were washed with 10 ml DPBS (Gibco) using 10 kDa MWCO filtration columns (Amicon). Fractions 6 and 12 of a sucrose gradient without OMVs was also washed with 10 ml DPBS and used as a blank for NanoSight analysis of their corresponding fraction, while DPBS was used as a blank for heterogeneous OMVs. NanoSight particle analysis was performed in 60 s reads in triplicate, with the gain set to 10, focus to −112, and camera level 8. Background from the corresponding blank samples was subtracted from each sample read and the average of the three reads was calculated and plotted as particle size versus number of particles per ml.

### Proteomic Analysis of OMVs

Proteomic analysis of OMVs was performed as described previously (44), using a pool of three biological OMV replicates. In brief, heterogeneous or fractionated OMV preparations (10 µg) were separated using Novex^®^ 10–20% Tris-Glycine gels (Life Technologies, CA, USA). Proteins contained within OMVs were visualized by staining with Coomassie Blue (Expedeon Ltd., Cambridgeshire, UK). For proteomic analyses, OMV preparations (6 µg) were reduced in 2.5 mM DTT followed by alkylation with 10 mM iodoacetamide and then 0.5 µg trypsin in 20 mM. Ammonium bicarbonate was added and the samples were incubated at 37°C overnight. Tryptic digests were analyzed by LC–MS/MS using the QExactive mass spectrometer (Thermo Scientific, Bremen, Germany) coupled online with an RSLC nano HPLC (Ultimate 3000, Thermo Scientific, Bremen, Germany) as previously described (44). Peptides were selected for MS/MS analysis in Full MS/dd-MS^2^ (TopN) mode with the following parameter settings: TopN 10, resolution 17500, MSMS AGC target 1e5, 60 ms Max IT, NCE 27, and 3 *m/z* isolation window. Underfill ratio was set at 10% and dynamic exclusion was set to 15 s. Data were processed using Proteome Discoverer V1.4 (Thermo Fisher Scientific) and searched against a custom database downloaded from the National Centre for Biotechnology Information ftp site using the MS Amanda search engine. The following search parameters were used: missed cleavages, 1; peptide mass tolerance, ±15 ppm; peptide fragment tolerance, ±0.2 Da; peptide charge, 2+, 3+, and 4+; static modifications, carbamidomethyl; and dynamic modification, oxidation (Met). Low and medium confidence peptides were filtered with at least 0.02 FDR (high confidence).

### Statistical Analysis

Error bars indicate the mean ± SEM. Fluorescence microscopy experiments were analyzed by One-Way Analysis of Variance (ANOVA) followed by Dunnett’s *post hoc* test. IL-8 responses were analyzed using ANOVA and compared to OMV non-treated group. Statistical analyses were performed using Prism software. Differences were considered significant when *P < 0.05, **P < 0.01, ***P < 0.001, ****P < 0.0001.

## Results

### A Heterogeneous Population of *H. pylori* OMVs Enter Host Cells by Micropinocytosis, Clathrin, and Caveolin-Dependent Endocytosis to Induce the Production of IL-8

Outer membrane vesicles enter non-phagocytic human epithelial cells to subsequently mediate a pro-inflammatory innate immune response. In this study, we sought to elucidate the mechanisms used by *H. pylori* OMVs to enter human epithelial cells and subsequently induce the production of IL-8. To do this, we initially blocked the clathrin, caveolin, or micropinocytosis pathways in both human gastric (AGS) and embryonic kidney (HEK293) cells using chemical inhibitors. We subsequently confirmed the viability of AGS and HEK293 cells post treatment with each chemical inhibitor, in addition to the inhibitors effectiveness (Figure S1 in Supplementary Material). To do this, both AGS and HEK293 cells were treated for 30 min with either: cytochalasin D or nocodazole, to block macropinocytosis, dynasore monohydrate to inhibit dynamin-dependent endocytosis which is utilized by both clathrin and caveolin-mediated entry, or valinomycin to block clathrin-mediated endocytosis (45). The viability of inhibitor-treated and control AGS and HEK293 cells was determined using the CellTiter-Glo assay (Figures S1A,B in Supplementary Material). Both AGS and HEK293 cells remained viable 4 h post treatment with each of the specific inhibitors of the macropinocytosis or endocytosis pathways, compared to azide control cells (Figure S1 in Supplementary Material). The specificity and effectiveness of each of the endocytosis inhibitors was next confirmed. To do this, AGS cells were pre-treated with each of the inhibitors prior to incubation with fluorescently labeled control compounds known to enter host cells via specific pathways. We found that the entry of fluorescently labeled human transferrin, which enters host cells specifically *via* clathrin-mediated endocytosis (42), was markedly reduced in cells pre-treated with dynasore or valinomycin as expected, to comparable levels as the positive control chlorpromazine (Figure S2A in Supplementary Material). Similarly, treatment of AGS cells with cytochalasin D reduced the internalization of micropinocytosis-dependent dextran70 into cells, and treatment with nocodazole resulted in a slight reduction of Dex70 into host cells (Figure S2B in Supplementary Material). Collectively, these findings confirmed the viability of both AGS and HEK cells post treatment with each inhibitor, and the effectiveness of each inhibitor in our assays.

We next sought to elucidate the endocytic mechanisms utilized by fluorescently labeled *H. pylori* OMV to enter host cells. However, this required us to ensure that we could remove any extracellular OMV-associated fluorescence from our analysis by quenching using the cell impermeant dye trypan blue. The effectiveness of trypan blue quenching of extracellular OMV-associated fluorescence was determined using AGS cells that had been cultured with DiO labeled *H. pylori* OMVs, then permeabilized using Triton-X and treated with trypan blue. Effective quenching of fluorescence was confirmed and quantified using both confocal microscopy and flow cytometry. We found that there was a slight reduction in extracellular fluorescence when OMV-stimulated AGS cells were incubated with trypan blue compared to stimulated cells that were not treated with trypan blue (Figure S3E in Supplementary Material). This suggests that there were very few extracellular OMVs present post incubation and subsequent sample preparation for analysis by flow cytometry. We also showed that OMV-stimulated AGS cells that were permeabilized and treated with trypan blue had negligible detectable fluorescence, compared with OMV-stimulated control cells (Figure S3 in Supplementary Material).

**Figure 1:**
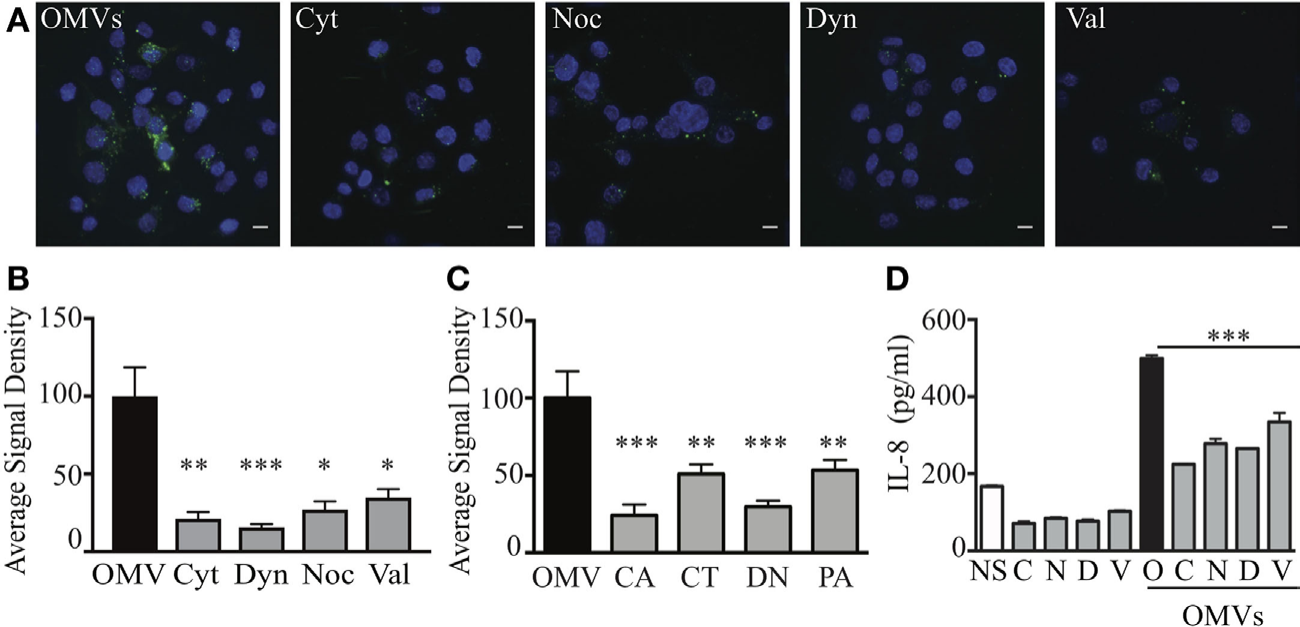
Heterogeneous sized outer membrane vesicles (OMVs) enter host cells *via* macropinocytosis, clathrin, caveolin, and dynamin-dependent endocytosis. **(A,B)** AGS cells were treated with cytochalasin D (Cyt), nocodazole (Noc), dynasore (Dyn), valinomycin (Val), or left untreated (OMV), prior to co-culture with DiO (green) labeled heterogeneous populations of OMVs. Nuclear DNA was stained with DAPI (blue) to allow enumeration of cells. Extracellular fluorescence was quenched with trypan blue. **(C)** The average signal density of internalized green fluorescent OMVs (OMVs) into cells pre-treated with siRNAs to specifically inhibit caveolin (CA), clathrin (CT), dynamin (DN), or macropinocytosis (PA) was measured and normalized to OMV alone group treated with control siRNA. **(D)** IL-8 production in AGS cells that were non-stimulated (NS, open bars), stimulated with OMVs alone as a control (black bar), or pre-treated with chemical inhibitors cytochalasin D (C), nocodazole (N), dynasore (D), valinomycin (V) (gray bars) prior to co-culture with OMVs. Data are represented as mean ± SEM of three replicate experiments. Line indicates statistical significance compared to OMV control group. Images are representative of three-independent experiments in which >100 cells were counted per treatment **(A)**, or pooled from three-independent experiments **(B–D)**. Error bars indicate ±SEM of >100 cells. *P < 0.05, **P < 0.01, ***P < 0.001.

Using these validated inhibitors of endocytosis and micropinocytosis pathways, we examined the endocytic mechanisms utilized by a fluorescently labeled heterogeneous population of *H. pylori* OMVs to enter host cells. AGS cells were pre-treated with each inhibitor prior to the addition of a heterogeneous population of fluorescently labeled OMVs and any extracellular fluorescence associated with OMVs was quenched using the cell impermeant dye trypan blue. The addition of fluorescently labeled OMVs to AGS cells pre-treated with inhibitors of endocytosis revealed that the transient inhibition of micropinocytosis, clathrin, caveolin, and dynamin-dependent endocytosis in AGS cells significantly reduced the amount of OMV-associated intracellular fluorescence, compared to untreated cells stimulated with OMVs (Figures 1A,B). We found that inhibition of dynamin had the greatest effect in reducing OMV entry into AGS cells (*P* > 0.001). Also, dynamin had a greater effect of inhibiting OMV entry into host cells when compared to valinomycin (*P* < 0.001) and nocodazole (*P* < 0.05). These findings indicate that a heterogeneous population of *H. pylori* OMVs enters AGS cells *via* all pathways of micropinocytosis and endocytosis, and with inhibition of dynamin having the greatest effect on reducing OMV entry into epithelial cells.

We next confirmed the ability of a heterogeneous population of *H. pylori* OMVs to enter AGS cells *via* multiple pathways of endocytosis, with a preferential use for dynamin-mediated entry using siRNA. To do this, we used siRNA to knockdown macropinocytosis, clathrin, or caveolin-mediated endocytosis in AGS cells prior to the stimulation of these cells with fluorescently labeled OMVs (Figure 1C). As a control, AGS cells were transfected with control siRNA. The efficiency of siRNA knockdown of each endocytosis or micropinocytosis pathway in AGS cells was confirmed only using qRT-PCR (Figure S4 in Supplementary Material). AGS cells in which micropinocytosis, clathrin, or caveolin-dependent endocytosis pathways were knocked down had a significant reduction in intracellular OMV-associated fluorescence compared to siRNA control cells stimulated with OMVs (Figure 1C). In particular, siRNA inhibition of caveolin and dynamin had the greatest effects at inhibiting OMV entry (*P* < 0.001) when compared to control siRNA stimulated cells, thus confirming our findings using chemical inhibition (Figure 1A), Furthermore, siRNA inhibition of caveolin and dynamin had the greatest effect of inhibiting OMV entry into AGS cells when compared to siRNA inhibition of clathrin and micropinocytosis (*P* < 0.05). Collectively, these findings identify that a heterogeneous population of OMVs enter AGS cells *via* micropinocytosis, clathrin, and caveolin-mediated endocytosis, with a preference for dynamin-dependent and caveolin-mediated endocytosis.

Numerous studies have reported that the internalization of OMVs into non-phagocytic epithelial cells results in the production of the pro-inflammatory cytokine, IL-8 (11, 39, 46). Therefore, we investigated if the inhibition of OMV entry into host cells *via* micropinocytosis and endocytosis also reduced IL-8 production by AGS and HEK293 cells. Pre-treatment of AGS cells with each of the chemical inhibitors significantly reduced IL-8 production in response to OMV stimulation (Figure 1D, *P* < 0.001). Similarly, inhibition of endocytosis and micropinocytosis pathways inhibited IL-8 production by HEK293 cells in response to OMV stimulation (Figure S5 in Supplementary Material). Collectively, these findings demonstrate that inhibition of each of these pathways significantly reduces OMV-mediated IL-8 responses in host cells.

### OMVs Size Determines Their Mechanism of Entry Into Host Cells

**Figure 2:**
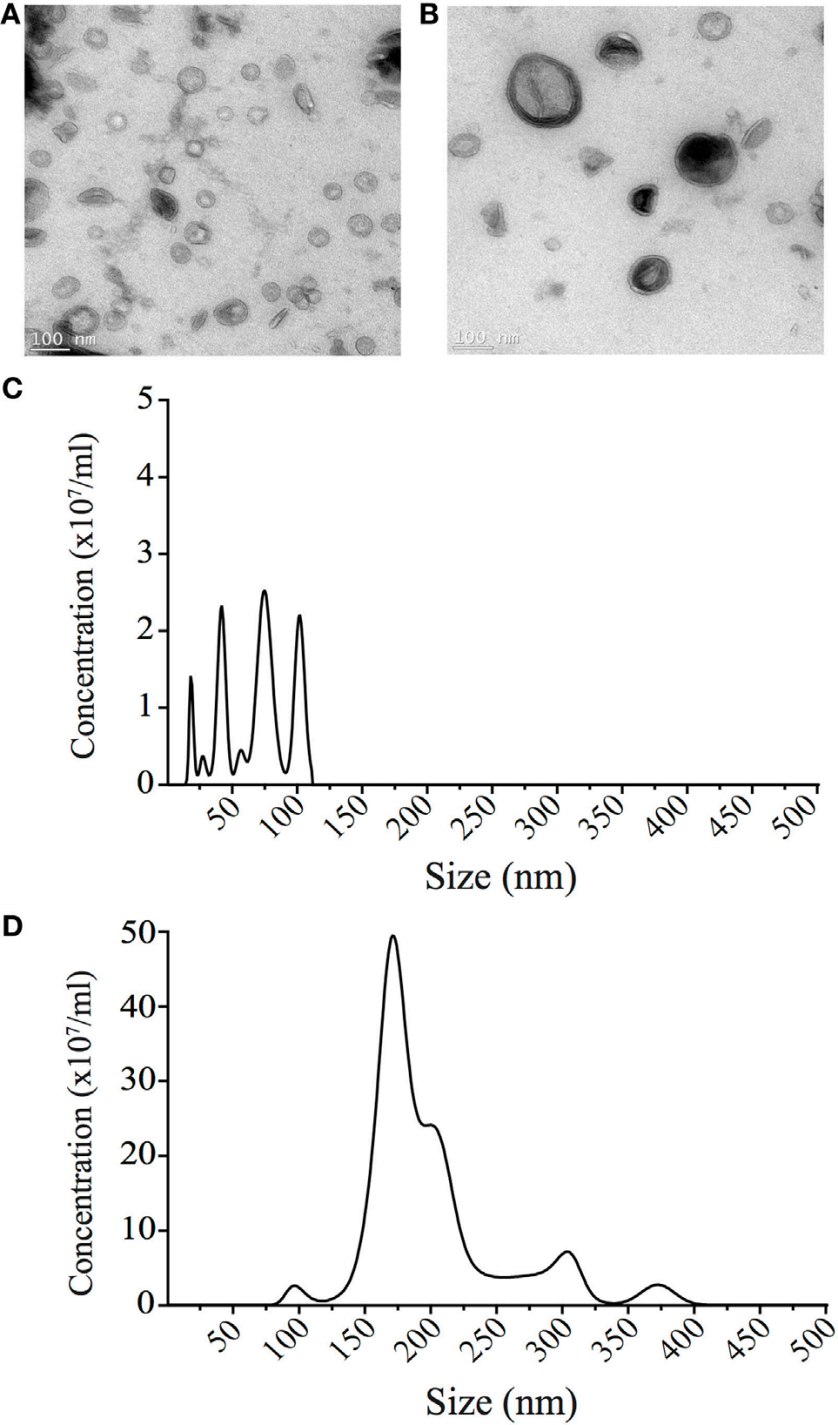
Small and large outer membrane vesicle (OMV) populations purified from a heterogeneous population of OMVs using sucrose gradient purification. **(A)** Transmission electron micrographs of small OMVs present in fraction 6 and **(B)** larger OMVs present in fraction 12. Scale bar represents 100 nm. **(C)** The sizes (nm) of OMVs found within fraction 6 containing small OMVs were determined using NanoSight Tracking Analysis and revealed four populations of OMVs ranging from 20 to 100 nm in size. **(D)** The size (nm) of OMVs found within fraction 12 containing larger OMVs was determined using NanoSight Tracking Analysis. Multiple populations of OMVs were contained within fraction 12, that ranged from 90 to 400 nm in size.

We next investigated the unknown role of OMV size on regulating the route of entry into host epithelial cells. To do this, we used sucrose gradient ultracentrifugation to separate a heterogeneous population of *H. pylori* OMVs ranging from 20 to 500 nm in size into two main populations, differing in both size and density (11). Analysis of the initial heterogeneous population using NanoSight Tracking Analysis revealed that approximately 96% of OMVs contained within the heterogeneous population were greater than 100 nm in diameter, with only 4% of OMVs being less than 100 nm in diameter (data not shown). Using sucrose density separation, OMVs were purified from fractions 6 and 12, which we have previously reported to contain small or large OMVs, respectively (11). The size of OMVs contained within fractions 6 and 12 were determined using NanoSight Tracking Analysis and were visualized using transmission electron microscopy (Figure 2). NanoSight analysis revealed that fraction 6 contained OMVs ranging between 20 and 100 nm in size, whereas OMVs purified from fraction 12 ranged from 90 to 450 nm in diameter (Figures 2C,D). Furthermore, there were multiple sized populations contained both within fractions 6 and 12 OMVs indicating that there is heterogeneity in the size of OMVs contained within these fractions (Figures 2C,D).

**Figure 3:**
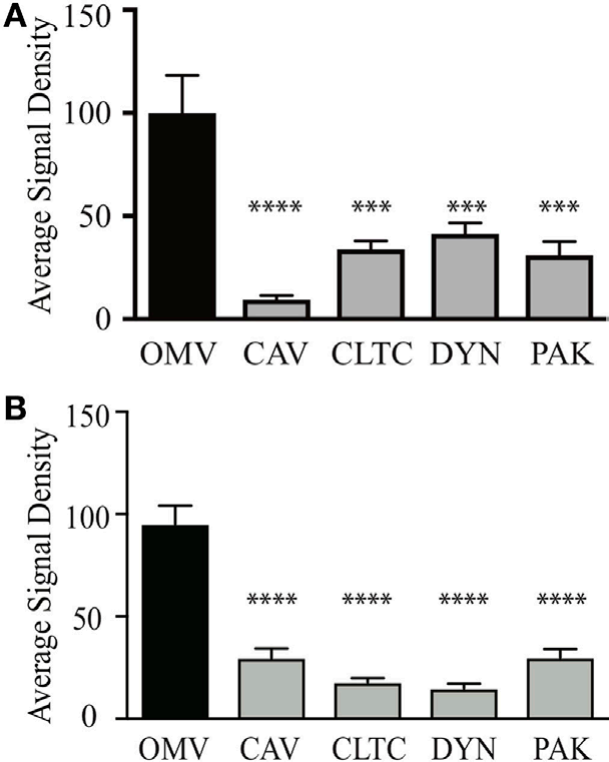
Outer membrane vesicle (OMV) size determines their route of entry into epithelial cells. The average signal density of internalized small **(A)** or large **(B)** green fluorescent (OMVs) into AGS cells pre-treated with siRNAs to specifically inhibit clathrin (CTLC), caveolin (CAV), dynamin (DYN), or macropinocytosis (PAK) was measured and normalized to OMV alone group treated with control siRNA. Data are pooled from three-independent experiments in which >100 AGS cells were counted per treatment. Error bars indicate ± SEM of >100 cells. ***P < 0.001, ****P < 0.0001.

We next determined whether OMV size may define the mechanism of entry into non-phagocytic host cells. To do this, OMVs from the small fraction (fraction 6) or large fraction (fraction 12) were added to AGS cells in which the micropinocytosis, clathrin, or caveolin-mediated endocytosis pathways were knocked down using siRNA. The number of internalized OMVs was subsequently quantified using confocal microscopy (Figure 3). Our findings revealed that although small OMVs could enter AGS cells *via* all pathways of micropinocytosis and endocytosis, they predominantly entered *via* caveolin-dependent endocytosis with the greatest efficiency (*P* < 0.0001, all compared to control OMVs) (Figure 3A). Comparison of OMV entry between all siRNA knockdown groups revealed that caveolin had the greatest effect at inhibiting entry of small OMVs into AGS cells compared to clathrin and dynamin (*P* < 0.01, *P* < 0.05, respectively).

In comparison, larger OMVs entered AGS cells *via* all pathways of micropinocytosis and endocytosis when compared to OMV control group (Figure 3B). Further comparisons between siRNA knockdown groups revealed that clathrin and dynamin had a greater effect at inhibiting the entry of large OMVs into AGS cells compared to caveolin and macropinocytosis [*P* < 0.01 dynamin Vs. macropinocytosis (PAK), *P* < 0.05 for all other analyses]. Collectively, these findings identify that although small and large OMVs enter host cells *via* all pathways of endocytosis, OMV size does determine their efficiency to enter host cells as caveolin has the greatest role in mediating entry of smaller OMVs into AGS cells. Also, larger OMVs may have a preference for clathrin and dynamin-mediated entry into host cells.

### OMV Size Determines Their Protein Content

**Figure 4:**
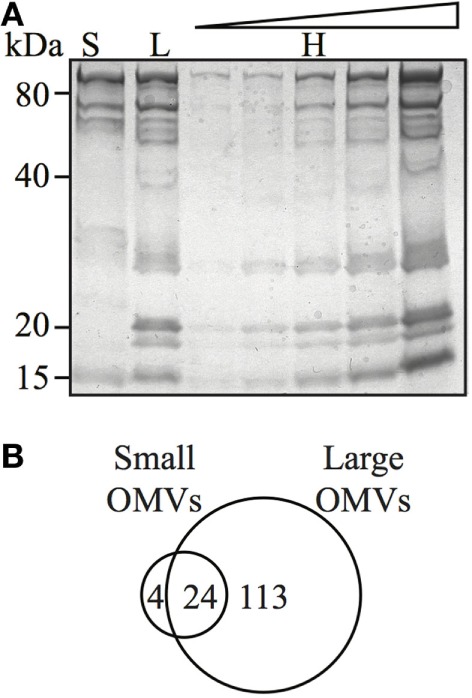
Outer membrane vesicle (OMV) size determines their protein content and composition. **(A)** Coomassie blue-stained polyacrylamide gel showing protein profiles of small (S), large (L), and heterogeneous (H) OMVs, the latter loaded in increasing concentration. **(B)** Venn diagram of proteins detected within small and large OMVs. A total of 137 proteins were detected in large OMVs and 28 proteins were detected in small OMVs. There were 24 proteins found in both large and small OMV populations.

Although bacteria may selectively package protein cargo into OMVs (27, 47, 48), the role of OMV size on regulating protein content and composition has not been investigated. Therefore, we sought to determine if *H. pylori* OMV size regulated their protein composition and cargo. For this, we initially examined small (fraction 6), large (fraction 12), and heterogeneous *H. pylori* OMV populations by SDS-PAGE (Figure 4A). We identified that the smaller *H. pylori* OMVs found within fraction 6 contained fewer proteins, compared to both larger OMVs contained within fraction 12 and the heterogeneous population of parent OMVs (Figure 4A). These findings support our previous preliminary findings identifying that fewer proteins were contained within smaller OMVs (11).

We further elucidated the role of OMV size on protein composition by performing detailed LC–MS/MS proteomic analysis of equivalent protein concentrations of small and large OMVs. LC–MS/MS proteomic analysis revealed that only a total of 28 *H. pylori*-specific proteins were contained within small OMVs isolated from fraction 6, compared with a total of 137 proteins contained within the large OMVs of fraction 12 (Figure 4B; Tables S1 and S2 in Supplementary Material, respectively). Of all of the proteins identified within both small and large OMVs, 113 were unique to large OMVs, 4 proteins were unique to small OMVs, and 24 proteins were common to both sized OMV preparations (Figure 4B). Interestingly, proteins associated with *H. pylori* survival or virulence were common to both small and large OMVs, including: urease A and B subunits, neutrophil activating protein, vacuolating cytotoxin (VacA), and the porin HopA (Table 1). Larger OMVs contained many of the known *H. pylori* adhesins, such as SabA, BabA, iron-regulated proteins, the Hop family of outer membrane proteins and numerous flagella basal and hook proteins (Table S2 in Supplementary Material). These proteins were absent in smaller OMVs (Table S1 in Supplementary Material). The four proteins exclusively contained within small OMVs were predominately associated with metabolism, and not virulence or adhesion (Table S1 in Supplementary Material). Collectively, this proteomic analysis revealed that larger OMVs contain significantly more proteins compared to smaller OMVs, and that most of the *H. pylori* adhesins are associated within larger OMVs purified from fraction 12. Furthermore, both small and large populations of OMVs contained many known virulence determinants, suggesting that both small and large sized OMVs play a role in mediating pathogenesis in the host.

**Table 1:** *Helicobacter pylori* proteins common in both small and large outer membrane vesicles (OMVs) (fractions 6 and 12).

Description	Gene	Score	Coverage	Score	Coverage
	
	No.	Small OMVs	Small OMVs	Large OMVs	Large OMVs
**Outer membrane proteins**					
Outer membrane protein HopA (Omp6)	HP0229	125.13	2.90	12,690.51	17.18
Thioredoxin	HP1548	166.95	14.42	1,183.40	34.62
Peptidoglycan-associated lipoprotein precursor (Omp18)	HP1125	205.39	10.61	1,809.68	17.88

**Metabolism**					
Urease subunit alpha	HP0073	13,062.19	56.72	68.49	8.40
Gamma-glutamyltranspeptidase	HP1118	693.31	5.82	7,237.48	16.93
Urease subunit beta	HP0072	26,367.78	52.55	2,594.83	14.76
Iron(III) ABC transporter periplasmic iron-binding protein (CeuE)	HP1562	363.55	7.21	6,586.97	37.24
Carbonic anhydrase	HP1186	1,372.04	18.81	5,111.16	34.65
Catalase-like protein	HP0485	123.63	4.14	3,216.80	30.89
Iron(III) ABC transporter periplasmic iron-binding protein (CeuE)	HP1561	140.59	2.99	2,559.96	18.21
Catalase	HP0875	7,584.68	47.13	26,552.56	54.26

**Post translational modification, protein turnover, chaperones**					
Chaperonin GroEL	HP0010	5,672.37	35.35	373.71	4.58
Bifunctional methionine sulfoxide reductase A/B protein	HP0224	1,826.54	14.48	12,539.26	31.20
Serine protease (HtrA)	HP1019	141.34	2.48	6,565.50	25.51
Alkyl hydroperoxide reductase (TsaA)	HP1563	503.19	5.56	413.07	14.65

**Other**					
Neutrophil activating protein (NapA) (bacterioferritin)	HP0243	4,741.96	38.19	376.22	16.67
Hypothetical protein HP0231	HP0231	400.83	3.77	8,592.37	38.49
Hypothetical protein HP0305	HP0305	142.05	5.98	2,442.19	38.04
Hypothetical protein HP1454	HP1454	234.35	4.95	9,609.87	35.64
Hypothetical protein HP0129	HP0129	293.54	7.09	3,695.28	24.82
Hypothetical protein HP0721	HP0721	683.38	18.42	6681.98	19.08
Vacuolating cytotoxin (VacA)	HP0887	1,510.21	3.33	7,883.58	15.89
Neuraminyllactose-binding hemagglutinin homolog (HpaA)	HP0410	227.63	4.82	4,096.93	14.86
Hypothetical protein HP1286	HP1286	613.71	11.54	4,171.54	17.58

## Discussion

Outer membrane vesicles are produced by all Gram-negative bacteria as part of their normal growth and have been reported to play a role in pathogenesis, bacterial cell communication, and biofilm formation [reviewed in Ref. (1)]. Furthermore, due to the highly inflammatory nature of OMVs, and their ability to harbor a range of bacterial proteins and immunogenic epitopes, they are extensively being developed as novel vaccine technology suitable for human and animal use [reviewed in Ref. (27)]. One of the key mechanisms whereby OMVs from various bacteria mediate an inflammatory response in the host is due to their ability to enter non-phagocytic host cells resulting in the production of pro-inflammatory cytokines (11, 29, 32, 33, 35–38, 49). Despite the numerous extensive studies investigating the mechanisms of OMV entry into host cells, the role of OMV size on mediating their mechanism of cellular entry and protein composition has not been determined.

The overall aim of this study was to elucidate the role of OMV size on determining their route of entry into non-phagocytic epithelial cells, in addition to defining their protein cargo and composition. To do this, we first examined the mode of entry of a heterogeneous population of *H. pylori* OMVs into host epithelial cells. Using chemical inhibition of the three main pathways of endocytosis: macropinocytosis, clathrin, and caveolin-mediated endocytosis, we found that a heterogeneous population of OMVs entered non-phagocytic human epithelial cells *via* all mechanisms of endocytosis and micropinocytosis, with chemical inhibition of dynamin-mediated endocytosis having the greatest effect in limiting OMV cellular entry (Figures 1A,B). These findings were validated by performing siRNA studies in which we confirmed the level of knockdown using qRT-PCR, but not at the protein level. Using siRNA to knockdown all three pathways of cellular entry, we confirmed our findings that OMVs entered host cells *via* micropinocytosis, clathrin, and cavolin-dependent endocytosis with inhibition of caveolin and dynamin having the greatest effects (Figure 1C). When examining the effect of inhibition of macropinocytosis, clathrin, and caveolin-dependent endocytosis on OMV-induced IL-8 responses, we discovered that all three pathways of cellular entry contributed to IL-8 production in response to OMV stimulation. This is the first report identifying that a small reduction in OMV cellular entry may have a profound effect on the level of the resulting host inflammatory response.

Particle size is known to play a role in determining the mechanism of endocytosis of lipid particles or latex beads into host cells (50, 51). In our previous study, we identified that OMVs less than 100 nm in diameter induced higher levels of NF-κB activity than larger OMVs, suggesting that these smaller OMVs may be more efficient at entering host epithelial cells and initiating pro-inflammatory responses (11). To determine the role of OMV size in host cell entry, we used our previously reported method to separate OMVs according to size and density (11). Using this method, we separated a heterogeneous population of *H. pylori* OMVs into two populations that were enriched for either small OMVs, up to approximately 100 nm in size, or large OMVs ranging between 90 and 400 nm (Figure 2). Using siRNA to limit OMV entry *via* micropinocytosis, clathrin, or caveolin-mediated endocytosis, we determined that small OMVs entered host cells *via* all three mechanisms (Figure 3) with a preference for caveolin-mediated entry. Whereas, siRNA studies determined that a population of larger OMVs entered host cells *via* all three mechanisms of endocytosis examined, and clathrin and dynamin may have the greatest effect at mediating entry (Figure 3). Collectively, these findings suggest that OMV size may regulate the route of entry into host cells, and that smaller OMVs preferentially enter non-phagocytic epithelial cells *via* caveolin (Figure 3). Previous studies have indicated that there are multiple mechanisms whereby bacterial OMVs can enter host cells. For example, Kesty et al. showed that enterotoxigenic *Escherichia coli* OMVs interacted with host cell caveolin, and that inhibition of clathrin-mediated endocytosis had no effect on vesicle uptake (32). However, clathrin-mediated endocytosis was reported by others to be required for internalization of *H. pylori* OMVs into host cells (29). Furthermore, there is some discrepancy in the literature regarding the specific mechanisms whereby OMVs from the same pathogen enter non-phagocytic host cells, and, therefore, the precise mode of OMV entry into host cells remains unclear. Based on our findings, we suggest that determining the size of OMVs contained within an OMV preparation is vital and may account for the differences seen in the modes of OMV entry between research groups.

In addition, this study identified a previously unknown role of OMV size in regulating OMV protein cargo composition. Specifically, proteomic analyses of small and large OMVs revealed that smaller OMVs contained significantly fewer proteins within them, compared to larger OMVs. Moreover, we showed that larger *H. pylori* OMVs contained bacterial adhesion proteins that were absent from smaller OMVs, which may facilitate their entry into host cells *via* receptor-mediated endocytosis. We identified 24 proteins common to both small and large OMVs; these were mostly proteins associated with virulence, including the vacuolating toxin (VacA), demonstrating a potential pathogenic role for OMVs of various sizes. An earlier study reported that OMVs containing VacA were less dependent on clathrin for entry, when compared with VacA negative OMVs, indicating that toxin containing OMVs may enter host cells by more than one mechanism (29). To our knowledge, no studies have been performed regarding the amount of VacA toxin associated with OMVs from different strains of *H. pylori*, or OMVs of different sizes, and it is plausible that different sized OMVs may contain varying amounts of toxin, which may also facilitate their entry *via* receptor-mediated endocytosis. This finding that variation in OMV size and cargo composition may regulate the mechanism of OMV-mediated endocytosis used to enter host cells warrants further investigation and forms the basis of future studies.

Collectively, our findings identify that OMV size has a key role in regulating both the route of OMV entry into host cells and their protein cargo composition. These findings highlight an important issue within the OMV field, being the importance of defining the size and composition of OMVs when determining their route of cellular entry and subsequent biological functions, as variability in OMV size and composition may alter the experimental outcomes. We propose variations in OMV size may be a reason for the discrepancies in the mechanisms of OMV host cell entry reported by various groups when examining OMVs from the same organisms, in addition to discrepancies in proteomic data. Therefore, we conclude that OMV size predetermines their route of cellular entry and their cargo composition. These findings have fundamental and significant implications that should be considered when examining the role of OMVs in pathogenesis, their protein content, and ultimately their use as vaccines against bacterial infections in humans. Further research elucidating the mechanisms whereby OMV size and composition regulates the mechanism(s) of OMV entry is vital to further develop OMVs as innovative vaccine technology, in addition to understanding their contribution to pathogenesis in the host.

## Author Contributions

LT, NB, DS, CL, KD, GR, and MK-L performed the research. MS, AH, and RF provided reagents and advice. LT, NB, and MK-L wrote the manuscript.

## Conflict of Interest Statement

The authors declare that the research was conducted in the absence of any commercial or financial relationships that could be construed as a potential conflict of interest. The reviewer KM and the handling Editor declared their shared affiliation.
