# Supplementary material for: Helicobacter pylori Outer Membrane Vesicle Size Determines Their Mechanisms of Host Cell Entry and Protein Content
Source: Front Immunol. 2018 Jul 2;9:1466. doi: 10.3389/fimmu.2018.01466 (PMC6036113; doi:10.3389/fimmu.2018.01466)
Supplement: Supplementary file 1 [file image_1.PDF]

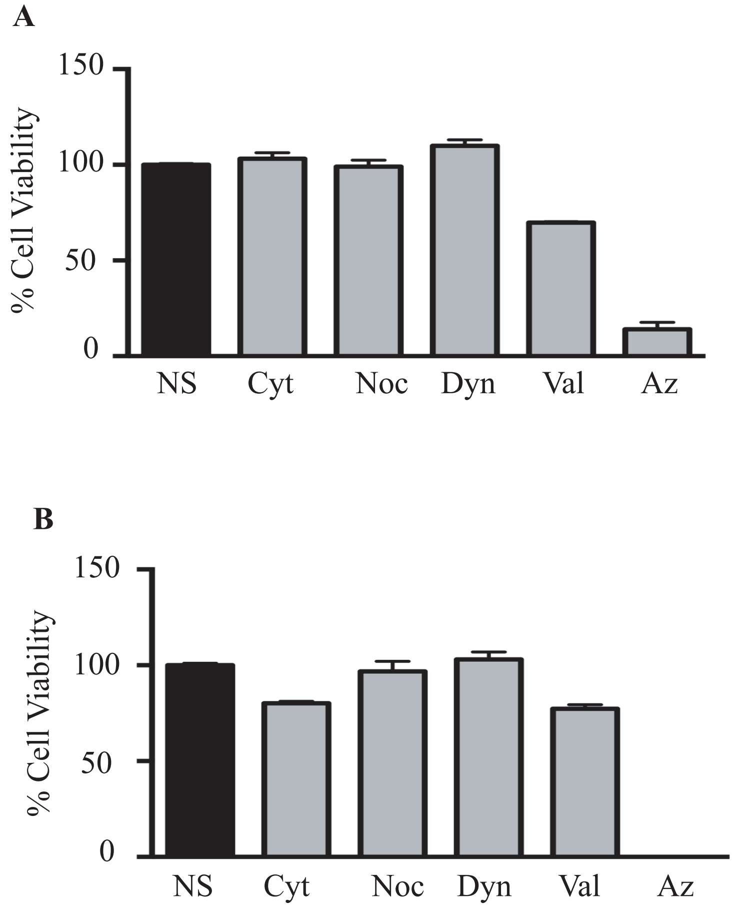

Supplementary Figure 1: AGS and HEK cells treated with inhibitors of endocytosis remain viable. AGS **(A)** and HEK (**B)** cells were treated with either of the inhibitors cytochalasin D (Cyt), dynasore (Dyn), nocodazole, (N), valinomycin (Val) or sodium azide (Az) as a positive control of cell death, for 30 minutes. Cells were subsequently washed and further incubated for 4 hours. Cell Viability was measured using the CellTiter-Glo assay. Percentage of cell viability was determined by comparing treated cells to non-treated control cells. Data are representative of two individual experiments. Data are represented as mean ± SEM.
