# Supplementary material for: Helicobacter pylori Outer Membrane Vesicle Size Determines Their Mechanisms of Host Cell Entry and Protein Content
Source: Front Immunol. 2018 Jul 2;9:1466. doi: 10.3389/fimmu.2018.01466 (PMC6036113; doi:10.3389/fimmu.2018.01466)
Supplement: Supplementary file 2 [file image_2.PDF]

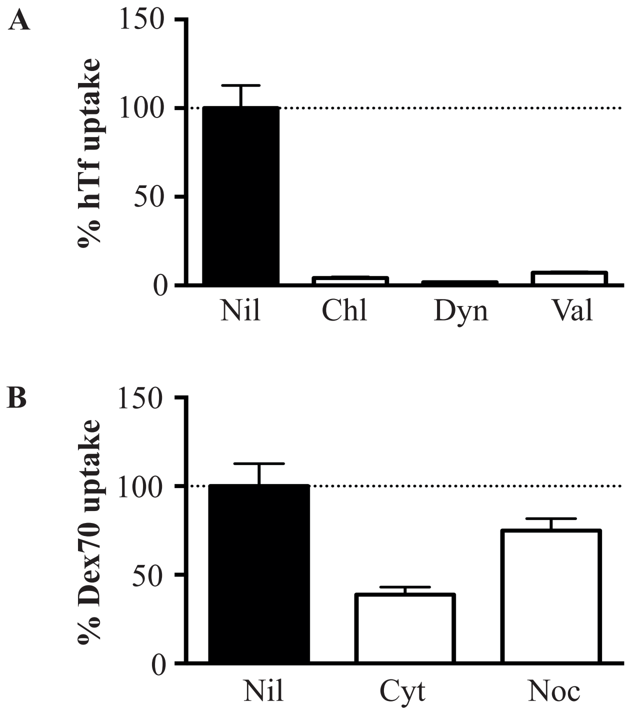

Supplementary Figure S2: Inhibitors of clathrin dependent endocytosis or macropinocytosis inhibit the uptake of hTf or Dex70, respectively.

(A) AGS cells were pre-treated with inhibitors of clathrin-dependent endocytosis; chlorpromazine, dynasore or valinomycin for 30 minutes prior to incubation with pHRODO red conjugated human transferrin (hTf) for 4 hours. The average signal density of internalised hTf was measured and normalized compared to the untreated control (Nil). Values are represented as % uptake compared to the Nil control. **(B)** AGS cells were pre-treated with inhibitors of macropinocytosis; cytochalasin D (CytD) or nocodazole (Noc) for 30 minutes prior to incubation with dextran fluorescein 70kDa (Dex70) for 4 hours. The average signal density of internalized Dex70 was measured and normalized compared to the untreated control (Nil). Values are represented as % uptake compared to the Nil control. Error bars indicate + SEM of > 100 cells.
