# Supplementary material for: Helicobacter pylori Outer Membrane Vesicle Size Determines Their Mechanisms of Host Cell Entry and Protein Content
Source: Front Immunol. 2018 Jul 2;9:1466. doi: 10.3389/fimmu.2018.01466 (PMC6036113; doi:10.3389/fimmu.2018.01466)
Supplement: Supplementary file 3 [file image_3.PDF]

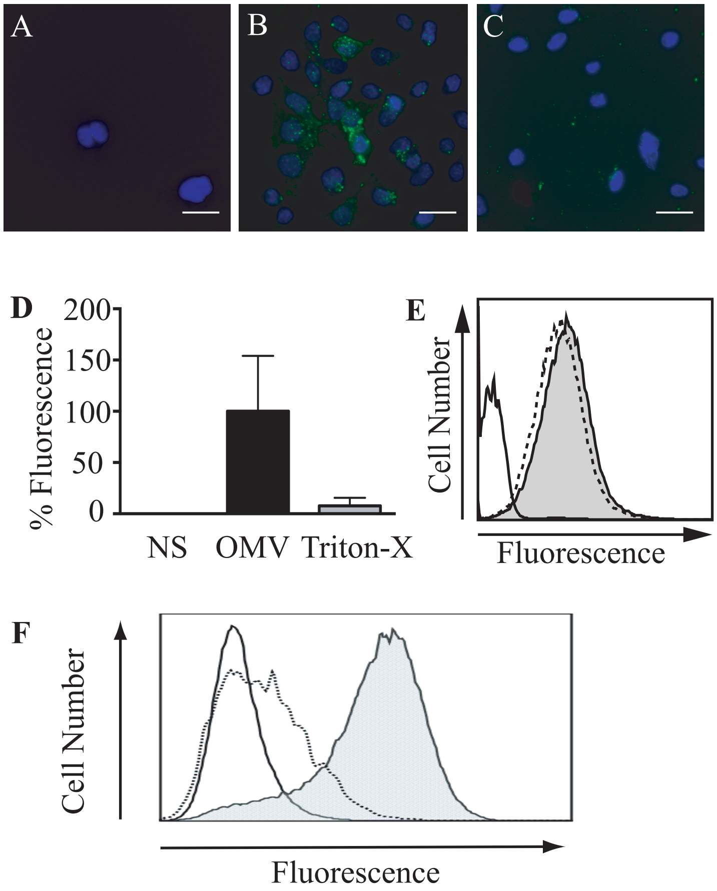

Supplementary Figure S3: OMV-associated fluorescence is effectively quenched using trypan blue. AGS cells were incubated with 50μg/ml of DiO fluorescently-labeled OMVs **(B, C)** or left non-stimulated **(A)**. Four hours later, cells were permeabilized with Triton-X **(C)** or left untreated **(A, B)** Scale bar indicates 10 μm. **(D)** The percentage of green fluorescence in samples A-C was quantified using confocal microscopy. The average signal density of DiO-OMV fluorescence (green) was determined. Values were normalised to those of the OMV group. Data are mean fluorescence in >100 cells per treatment. Error bars indicate ± standard errors of the mean (SEM). **(E)** AGS cells were incubated with 50μg/ml of DiO fluorescently-labeled OMVs (dotted line, solid line with shading) or non-stimulated as controls (solid line). Four hours later, cells were treated with trypan blue (dotted line) or left non-treated (solid line with shading). Cells were analysed by flow cytometry for their level of OMV-associated fluorescence. **(F)** AGS cells that were either non-stimulated (NS-black line), or stimulated with DiO-labeled OMVs (solid line with shading, dotted line). OMV-stimulated cells either treated with Triton-X (dotted line) or left untreated (solid line with shading) prior to the addition of trypan blue to quench OMV-associated fluorescence. Cells were analysed by flow cytometry for their level of OMV-associated fluorescence.
