# Supplementary material for: Helicobacter pylori Outer Membrane Vesicle Size Determines Their Mechanisms of Host Cell Entry and Protein Content
Source: Front Immunol. 2018 Jul 2;9:1466. doi: 10.3389/fimmu.2018.01466 (PMC6036113; doi:10.3389/fimmu.2018.01466)
Supplement: Supplementary file 4 [file image_4.PDF]

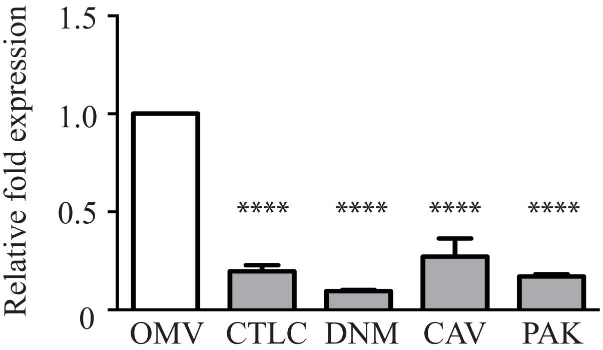

Supplementary Figure S4. Confirmation of siRNA knock-down of endocytosis pathways by qPCR. AGS cells were treated with siRNA to knock down clathrin-mediated endocysotis (CTLC), dynamin-dependent endocytosis (DNM), caveolin-mediated endocytosis (CAV) or macropinocytosis (PAK). Confirmation of knockdown was assessed by qPCR analysis using specific Taqman primers. Data are pooled from 4 experiments performed in triplicate. Bars indicate the mean of 4 expeiments and error bars represent the standard errors of the means, **** $p<0.0001$
