# Supplementary material for: Helicobacter pylori Outer Membrane Vesicle Size Determines Their Mechanisms of Host Cell Entry and Protein Content
Source: Front Immunol. 2018 Jul 2;9:1466. doi: 10.3389/fimmu.2018.01466 (PMC6036113; doi:10.3389/fimmu.2018.01466)
Supplement: Supplementary file 5 [file image_5.PDF]

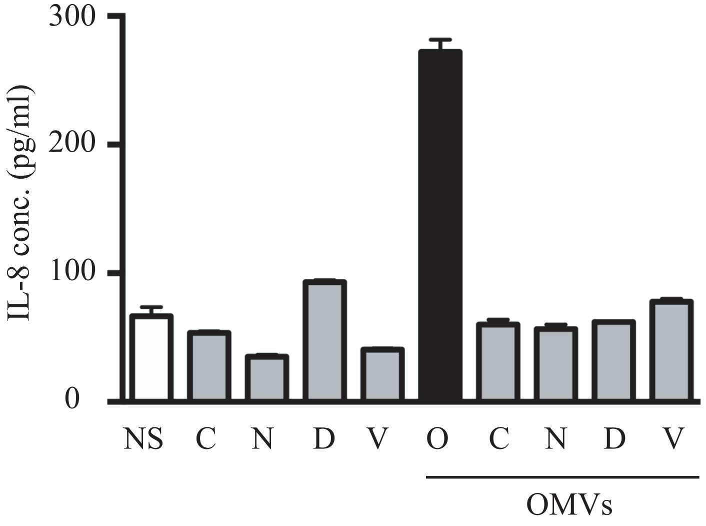

Supplementary Figure 5: Inhibition of OMV entry reduces IL-8 production by HEK293 cells.
IL-8 production in HEK293 cells that were non-stimulated (NS, open bars), stimulated with OMVs alone as a control (black bar) or pre-treated with chemical inhibitors cytochalasin D (C), nocodazole (N), dynasore (D), valinomycin (V) (grey bars) prior to co-culture with OMVs. IL-8 production was measured by ELISA. Error bars indicate mean + standard errors of the means (SEM) of technical replicates. Data are representative of 2 biological experiments.
