# Supplementary material for: Helicobacter pylori Outer Membrane Vesicle Size Determines Their Mechanisms of Host Cell Entry and Protein Content
Source: Front Immunol. 2018 Jul 2;9:1466. doi: 10.3389/fimmu.2018.01466 (PMC6036113; doi:10.3389/fimmu.2018.01466)
Supplement: Supplementary file 6 [file table_1.PDF]

TABLE S1 *H. pylori* OMV proteins detected in fraction 6

Accession #	Description	Gene No.	Score	Coverage	# of peptides	kDa	Calculated pI
	**Metabolism**						
NP_207952	flavodoxin (fldA)	HP1161	969.27	17.07	2	17.5	4.59
NP_207309	glutamine synthetase (glnA)	HP0512	1831.71	14.55	6	54.5	6.13
NP_207447	nonheme iron-containing ferritin (pfr)	HP0653	186.01	13.77	1	19.3	5.67
NP_206828	type II citrate synthase	HP0026	188.78	4.23	1	48.3	7.88
