# Supplementary material for: Helicobacter pylori Outer Membrane Vesicle Size Determines Their Mechanisms of Host Cell Entry and Protein Content
Source: Front Immunol. 2018 Jul 2;9:1466. doi: 10.3389/fimmu.2018.01466 (PMC6036113; doi:10.3389/fimmu.2018.01466)
Supplement: Supplementary file 7 [file table_2.PDF]

TABLE S2 *H. pylori* OMV proteins detected in fraction 12

Accession #	Description	Gene No.	Score	Coverage	# peptides	kDa	Calculated pI
**Major outer membrane protein family**							
NP_207500	outer membrane protein (omp15)	HP0706	2671.51	32.23	5	29.9	8.87
NP_207968	outer membrane protein (omp27)	HP1177	35410.35	30.11	13	69.6	9.11
NP_208292	outer membrane protein HorK (omp32)	HP1501	4858.85	24.74	6	42.9	9.41
NP_208260	outer membrane protein HorJ (omp31)	HP1469	3672.96	23.39	5	28.1	9.51
NP_207704	outer membrane protein AlpA, HopC (omp20)	HP0912	3424.75	22.14	5	55.9	9.10
NP_207025	outer membrane protein HopM (omp5)	HP0227	4680.15	19.83	8	75.7	8.40
NP_207516	outer membrane protein (omp16)	HP0722	10128.12	16.45	10	67.0	9.20
NP_207689	outer membrane protein BabB, HopT (omp19)	HP0896	7863.94	15.11	6	77.6	8.50
NP_207519	outer membrane protein SabA, HopP (omp17)	HP0725	3960.35	12.72	5	69.4	9.07
NP_207270	outer membrane protein HorE (omp11)	HP0472	402.79	12.37	2	20.9	9.13
NP_206927	outer membrane protein HorB (omp4)	HP0127	386.82	9.79	2	31.9	9.54
NP_207947	outer membrane protein HopI (omp25)	HP1156	3703.52	9.77	7	76.3	9.06
NP_207052	outer membrane protein HopG (omp8)	HP0254	1512.42	8.35	4	47.5	9.19
NP_207050	outer membrane protein HopF (omp7)	HP0252	494.92	8.21	2	53.3	9.26
NP_207465	outer membrane protein HorF (omp14)	HP0671	452.71	3.33	1	30.3	9.11
NP_207705	outer membrane protein AlpB, HopB (omp21)	HP0913	2869.39	2.65	1	57.0	9.17
NP_206827	outer membrane protein HopD (omp2)	HP0025	167.16	1.55	1	77.6	9.10
NP_207115	outer membrane protein HopU (omp9)	HP0317	5264.76	9.53	4	81.1	8.50
NP_206811	outer membrane protein HopZ	HP0009	3073.13	4.42	2	69.2	8.92
**Iron-regulated outer membrane proteins**							
NP_208303	iron-regulated outer membrane protein (frpB)	HP1512	5230.75	9.92	7	97.3	9.01
NP_207480	iron(III) dicitrate transport protein (fecA)	HP0686	409.44	4.30	2	87.6	9.47
NP_208191	iron(III) dicitrate transport protein (fecA)	HP1400	302.90	1.66	1	94.8	9.04
**Efflux pump outer membrane proteins and other outer membrane proteins**							
NP_207632	outer membrane protein P1 (ompP1)	HP0839	2280.71	11.75	6	63.6	9.47
NP_207449	protective surface antigen D15	HP0655	1821.64	6.33	5	102.6	9.00
NP_208247	membrane-associated lipoprotein (lpp20)	HP1456	9984.98	56.00	9	19.1	9.51
**Cell wall/membrane biogenesis**							

NP_207761	nickel-cobalt-cadmium resistance protein (nccB)	HP0970	341.93	6.41	2	39.6	8.56
NP_207401	membrane fusion protein (mtrC)	HP0606	3238.19	44.02	9	25.9	8.82
NP_208362	rare lipoprotein A (rlpA)	HP1571	1020.16	14.29	4	35.5	9.33
NP_208142	protease	HP1350	4719.19	26.80	13	50.5	9.38
**Cell motility**							
NP_207396	flagellin A FlaA	HP0601	761.89	8.04	3	53.3	6.43
NP_207699	flagellar basal body rod modification protein FlgD	HP0907	288.29	5.32	1	33.7	5.24
NP_208268	flagellar basal body P-ring biosynthesis protein FlgA	HP1477	155.72	3.21	1	24.3	8.97
NP_207664	flagellar hook protein FlgE	HP0870	4847.00	21.17	11	76.2	5.25
NP_208348	flagellar hook-basal body protein FliE	HP1557	722.41	20.18	2	12.2	8.40
**Intracellular trafficking and secretion**							
NP_206841	conjugal plasmid transfer system protein ComB9	HP0039	261.10	3.68	1	37.5	9.07
NP_207917	translocation protein TolB	HP1126	3380.35	25.18	8	47.8	9.14
**Posttranslational modification, protein turnover, chaperones**							
NP_208253	secreted protein involved in flagellar motility	HP1462	1744.55	30.23	4	20.5	7.03
NP_207175	thiol:disulfide interchange protein (dsbC)	HP0377	1743.55	15.84	3	25.3	8.12
NP_206974	cell binding factor 2	HP0175	6379.87	31.10	9	34.0	9.29
**Metabolism**							
NP_207732	amino acid ABC transporter periplasmic binding protein (yckK)	HP0940	1739.55	19.14	5	28.7	9.44
NP_207963	glutamine ABC transporter periplasmic glutamine-binding protein (glnH)	HP1172	1001.65	23.83	5	31.2	7.40
NP_207073	ATP-dependent nuclease (addB)	HP0275	2323.09	11.16	5	49.7	8.76
NP_208252	cytochrome c551 peroxidase	HP1461	789.36	12.29	3	38.8	9.03
NP_208355	predicted outer membrane lipoprotein plpA	HP1564	3116.99	26.57	6	30.1	9.10
NP_208019	cytochrome c553	HP1227	1869.16	31.25	3	10.3	9.48
NP_207121	nuclease NucT	HP0323	181.24	5.00	1	20.1	9.47
NP_206904	2',3'-cyclic-nucleotide 2'-phosphodiesterase	HP0104	169.13	1.72	1	65.8	8.68
**Relaxes negatively supercoiled DNA**							
NP_206916	DNA topoisomerase I	HP0116	104.24	1.90	1	83.1	8.90
**Other**							
NP_208058	NADH dehydrogenase subunit G	HP1266	195.29	1.18	1	94.2	5.44
NP_207617	thioredoxin (trxA)	HP0824	375.61	21.70	2	11.8	5.22
NP_207590	flagellar sheath adhesin hpaA	HP0797	12199.52	31.15	8	29.0	8.53

NP_207451	processing protease (ymxG)	HP0657	2028.30	20.83	9	48.8	9.10
NP_207802	protease (pqqE)	HP1012	4013.25	21.40	9	50.3	8.76
NP_207391	tumor necrosis factor alpha-inducing protein	HP0596	3433.69	44.27	6	21.9	8.51
NP_206820	hypothetical protein	HP0018	383.87	6.40	2	53.4	8.98
NP_207630	hypothetical protein HP0837	HP0837	1577.25	38.24	4	11.2	9.44
NP_207964	hypothetical protein HP1173	HP1173	3378.60	33.33	6	20.6	8.84
NP_206935	hypothetical protein HP0135	HP0135	940.29	31.82	1	5.0	7.18
NP_206897	hypothetical protein HP0097	HP0097	2268.08	28.69	5	26.1	8.47
NP_207283	hypothetical protein HP0486	HP0486	7848.20	27.27	12	59.4	9.33
NP_207002	hypothetical protein HP0203	HP0203	423.85	26.37	2	10.6	8.65
NP_206930	hypothetical protein HP0130	HP0130	1128.02	17.48	4	32.7	9.47
NP_207539	hypothetical protein HP0746	HP0746	1528.91	17.42	6	48.1	9.17
NP_208077	hypothetical protein HP1285	HP1285	1284.72	17.39	3	26.3	9.28
NP_207915	hypothetical protein HP1124	HP1124	987.19	16.92	4	38.4	9.36
NP_206930	hypothetical protein HP0130	HP0130	1128.02	17.48	4	32.7	9.47
NP_207539	hypothetical protein HP0746	HP0746	1528.91	17.42	6	48.1	9.17
NP_208077	hypothetical protein HP1285	HP1285	1284.72	17.39	3	26.3	9.28
NP_207915	hypothetical protein HP1124	HP1124	987.19	16.92	4	38.4	9.36
NP_208118	hypothetical protein HP1326	HP1326	357.66	15.20	2	13.8	9.63
NP_206887	hypothetical protein HP0087	HP0087	1291.71	14.00	6	52.3	9.23
NP_207033	hypothetical protein HP0235	HP0235	1597.57	13.80	4	39.4	7.53
NP_207453	hypothetical protein HP0659	HP0659	1280.98	13.77	5	47.6	8.94
NP_208248	hypothetical protein HP1457	HP1457	3099.03	12.86	3	23.2	8.59
NP_207513	hypothetical protein HP0719	HP0719	1613.86	12.84	1	12.2	9.99
NP_208314	hypothetical protein HP1524	HP1524	212.75	12.17	1	13.3	6.93
NP_207706	hypothetical protein HP0914	HP0914	1805.81	11.67	4	58.8	9.16
NP_207030	hypothetical protein HP0232	HP0232	1262.52	11.63	2	22.9	9.48
NP_207289	hypothetical protein HP0492	HP0492	1773.89	11.51	4	31.9	9.11
NP_206959	hypothetical protein HP0160	HP0160	1571.97	11.11	3	34.1	9.09
NP_208244	hypothetical protein HP1453	HP1453	3941.97	10.72	6	82.3	5.83
NP_208315	hypothetical protein HP1525	HP1525	937.75	10.43	2	24.8	9.48
NP_208246	hypothetical protein HP1455	HP1455	615.25	10.00	1	14.7	9.39
NP_207657	hypothetical protein HP0863	HP0863	1690.66	9.96	4	61.9	9.16

NP_208317	hypothetical protein HP1527	HP1527	1113.04	9.60	5	54.7	7.08
NP_207003	hypothetical protein HP0204	HP0204	305.30	9.45	1	14.5	9.11
NP_207874	hypothetical protein HP1083	HP1083	1992.68	9.39	4	53.1	9.47
NP_207206	hypothetical protein HP0408	HP0408	125.06	9.26	1	18.7	9.45
NP_207724	hypothetical protein HP0932	HP0932	172.54	9.00	1	11.9	8.79
NP_207908	hypothetical protein HP1117	HP1117	1543.22	8.98	2	29.0	8.50
NP_207305	hypothetical protein HP0508	HP0508	964.49	8.19	4	52.6	9.16
NP_206880	hypothetical protein HP0080	HP0080	1173.89	8.09	4	65.9	4.64
NP_207629	hypothetical protein HP0836	HP0836	665.53	7.56	1	13.2	10.17
NP_207400	hypothetical protein HP0605	HP0605	834.92	7.55	3	54.6	9.00
NP_207764	hypothetical protein HP0973	HP0973	1225.88	7.08	2	39.8	9.36
NP_207009	hypothetical protein HP0211	HP0211	97.57	6.80	1	27.3	8.31
NP_207872	hypothetical protein HP1081	HP1081	280.12	6.28	1	23.7	9.48
NP_207165	hypothetical protein HP0367	HP0367	416.30	5.94	1	23.2	8.22
NP_207504	hypothetical protein HP0710	HP0710	5878.12	5.91	4	73.3	7.56
NP_207889	hypothetical protein HP1098	HP1098	474.57	5.52	1	31.6	8.47
NP_207958	hypothetical protein HP1167	HP1167	725.63	5.52	2	52.6	9.50
NP_207574	hypothetical protein HP0781	HP0781	362.42	5.13	2	49.4	5.81
NP_207848	hypothetical protein HP1057	HP1057	315.39	5.00	1	27.4	9.35
NP_207007	hypothetical protein HP0209	HP0209	285.15	4.89	2	52.6	9.58
NP_208337	hypothetical protein HP1546	HP1546	142.61	4.71	1	19.6	8.50
NP_207846	hypothetical protein HP1055	HP1055	204.99	4.46	1	35.7	8.43
NP_207350	hypothetical protein HP0555	HP0555	156.67	3.30	1	31.4	9.25
NP_207847	hypothetical protein HP1056	HP1056	155.43	2.82	1	32.3	8.94
NP_207575	hypothetical protein HP0782	HP0782	204.75	2.20	1	52.0	9.31
NP_207897	hypothetical protein HP1106	HP1106	180.95	2.07	1	51.1	9.50
NP_207581	hypothetical protein HP0788	HP0788	148.52	1.80	1	56.8	8.95
NP_207745	hypothetical protein HP0953	HP0953	2854.88	46.81	8	21.2	8.60
NP_207520	hypothetical protein HP0726	HP0726	165.99	2.95	1	34.1	9.67
